# Evaluation of the need for dosing adaptations in obese patients for surgical antibiotic prophylaxis: a model-based analysis of cefazolin pharmacokinetics

**DOI:** 10.1016/j.bja.2024.11.044

**Published:** 2025-02-01

**Authors:** Davide Bindellini, Philipp Simon, David Busse, Robin Michelet, David Petroff, Linda B.S. Aulin, Christoph Dorn, Markus Zeitlinger, Wilhelm Huisinga, Hermann Wrigge, Charlotte Kloft

**Affiliations:** 1 Department of Clinical Pharmacy and Biochemistry, Institute of Pharmacy, Freie Universitaet Berlin, Berlin, Germany; 2 Graduate Research Training Programme, PharMetrX, Berlin, Germany; 3 Department of Anaesthesiology and Operative Intensive Care, Faculty of Medicine, University of Augsburg, Augsburg, Germany; 4 Integrated Research and Treatment Center (IFB), Adiposity Diseases, University of Leipzig, Leipzig, Germany; 5 Department of Anesthesiology and Intensive Care Medicine, University of Leipzig Medical Center, Leipzig, Germany; 6 Boehringer Ingelheim Pharma GmbH & Co. KG, Ingelheim am Rhein, Germany; 7 Clinical Trial Centre Leipzig, University of Leipzig, Leipzig, Germany; 8 Institute of Pharmacy, University of Regensburg, Regensburg, Germany; 9 Department of Clinical Pharmacology, Medical University of Vienna, Vienna, Austria; 10 Institute of Mathematics, University of Potsdam, Potsdam, Germany; 11 Bergmannstrost Hospital Halle, Department of Anaesthesiology, Intensive Care and Emergency Medicine, Pain Therapy, Halle, Germany; 12 Medical Faculty, Martin-Luther-University Halle-Wittenberg, Halle, Germany

**Keywords:** cefazolin, microdialysis, obesity, pharmacokinetics, population analysis, surgical antibiotic prophylaxis

## Abstract

**Background:**

Cefazolin is used as a prophylactic antibiotic to reduce surgical site infections (SSIs). Obesity has been identified as a risk factor for SSIs. Cefazolin dosing recommendations and guidelines are currently inconsistent for obese patients. As plasma and target-site exposure might differ, pharmacokinetic data from the sites of SSIs are essential to evaluate treatment efficacy: these data can be obtained via tissue microdialysis. This analysis was designed to evaluate the need for dosing adaptations in obese patients for surgical prophylaxis.

**Methods:**

Data from 15 obese (BMI_median_ = 52.6 kg m^−2^) and 15 age- and sex-matched nonobese patients (BMI_median_ = 26.0 kg m^−2^) who received 2 g cefazolin i.v. infusion for infection prophylaxis were included in the analysis. Pharmacokinetic data from plasma and interstitial space fluid (ISF) of adipose tissue were obtained and analysed simultaneously using nonlinear mixed-effects modelling. Dosing regimens were evaluated by calculating the probability of target attainment (PTA) and the cumulative fraction of response (CFR) for plasma and ISF using unbound cefazolin concentration above minimum inhibitory concentration 100% of the time as target (*f*T_>MIC_ = 100%). Dosing regimens were considered adequate when PTA and CFR were ≥90%.

**Results:**

Evaluation of cefazolin doses of 1 and 2 g with redosing at either 3 or 4 h by PTA and CFR in plasma and ISF found 2 g cefazolin with redosing at 4 h to be the most suitable dosing regimen for both obese and nonobese patients (PTA >90% and CFR >90% for both).

**Conclusions:**

This model-based analysis, using *f*T_>MIC_ = 100% as a target, showed that cefazolin dosing adaptations are not required for surgical prophylaxis in obese patients.

**Editor's key points**

• Perioperative dosing recommendations for cefazolin, used as a prophylactic antibiotic to reduce surgical site infections, are currently inconsistent for obese patients.
• Pharmacokinetic data for cefazolin from plasma and interstitial space fluid (ISF) of adipose tissue obtained by microdialysis for obese and nonobese surgical patients receiving prophylactic cefazolin were analysed simultaneously using nonlinear mixed-effects modelling.
• In the context of perioperative antibiotic prophylaxis, administration of 2 g cefazolin i.v. with redosing at 4 h was the most suitable dosing regimen for both obese and nonobese patients such that no dose adjustment is necessary.

Surgical site infections (SSIs) pose a significant risk to patient safety. However, the incidence of SSIs can be reduced by use of appropriate perioperative antibiotic prophylaxis.^1^^,^^2^ The incidence of SSI is higher in obese than in nonobese patients.^3^, ^4^, ^5^ Obesity is associated with atypical anthropometric values and pathophysiological alterations that can influence drug pharmacokinetics. These alterations include changes in body composition, tissue distribution, and renal and hepatic drug elimination.^6^, ^7^, ^8^, ^9^ Consequently, standard dosing regimens of antibiotics might not provide optimal drug exposure in obese patients, potentially compromising the effectiveness of perioperative antibiotic prophylaxis, highlighting the need for identifying optimal prophylactic dosing regimens for obese patients.

Cefazolin is commonly used for perioperative antibiotic prophylaxis owing to its activity against pathogens frequently encountered in SSIs, such as *Escherichia coli* and *Staphylococcus aureus*.^10^^,^^11^ Some studies have investigated the need for perioperative prophylactic cefazolin dosing adjustments for obese patients, but the results are contradictory.^12^ Although the majority of evidence supports standard dosing for cefazolin (2 g, short-term i.v. infusion), three out of four studies that applied modelling and simulation frameworks concluded the opposite.^13^, ^14^, ^15^ In addition, a second antibiotic dose is recommended after twice its half-life,^16^, ^17^, ^18^ which would be 3–4 h after the first dose in the case of cefazolin, but this recommendation has not been investigated conclusively.

For cefazolin, the pharmacokinetic/pharmacodynamic (PK/PD) target has been set to unbound drug concentration in plasma above minimum inhibitory concentration (MIC) 100% of the operation time.^19^ To evaluate the safety and effectiveness of perioperative antibiotic prophylaxis, it is also essential to measure drug concentrations at the site of potential infection (interstitial space fluid [ISF] of soft tissue),^20^^,^^21^ as drug exposure in plasma might not reflect exposure in the ISF. Unbound drug concentrations from the ISF can be obtained by microdialysis, a minimally invasive sampling technique to obtain target-site concentrations.^22^ Pharmacometric models, which allow simultaneous integration and analysis of data from different sources (e.g. measurements from plasma and ISF), can be used to simulate concentration–time profiles to investigate the influence of obesity on the probability of achieving a specific PK/PD target.

We aimed to evaluate the adequacy of clinically relevant cefazolin dosing regimens (i.e. dose and dosing intervals) for obese patients for perioperative antibiotic prophylaxis and to derive dosing recommendations by application of modelling and simulation techniques. We leveraged published PK data from obese and nonobese patients, integrating observed cefazolin concentrations in both plasma and ISF.

## Methods

This is a *post hoc*, exploratory analysis of a study that was approved by the Leipzig University Ethics Committee dated on July the 12th, 2013 (No. 121-13-28012013) and the Federal Institute of Drug and Medical Devices dated on May the 10th, 2013 (BfArM No. 4038808). It was registered in the EU Clinical Trials Register (EudraCT registration No. 2012-004383-22) and the German Clinical Trials Register dated on August the 27th, 2013 (registration No. DRKS00004776). The study was designed in accordance with the principles of the Declaration of Helsinki. Written informed consent was obtained from every enrolled participant.

### Study design and patient population

Details about the study design, sample size calculation, and inclusion and exclusion criteria have been described.^23^^,^^24^ Briefly, data were obtained from a prospective, controlled, single-centre, open-label clinical trial. During the trial, 15 obese patients (BMI ≥35 kg m^−2^) undergoing bariatric surgery and 15 nonobese patients (18.5 kg m^−2^ ≤ BMI ≤ 30 kg m^−2^) undergoing elective abdominal surgery were enrolled. The groups were matched according to participant age and sex.

All participants were administered a single dose of 2 g cefazolin by i.v. infusion over 30 min after induction of general anaesthesia. Blood samples to obtain plasma samples were collected at 0.5, 1, 2, 3, 4, 5, 6, and 8 h after the start of cefazolin infusion. Plasma samples collected at 0.5, 1, 4, and 8 h underwent ultrafiltration to measure unbound cefazolin concentration. Microdialysis catheters (CMA 63 microdialysis probe, cut-off 20 000 Da, membrane length 30 mm; CMA, Kista, Sweden) were inserted into the ISF of the subcutaneous adipose tissue of both upper arms (right and left) 90 min before cefazolin administration. Microdialysis samples (flow rate = 2 μl min^−1^) were collected in intervals of 0–0.5, 0.5–1, 1–1.5, 1.5–2, 2–3, 3–4, 4–5, 5–6, 6–7, and 7–8 h after the dose. Retrodialysis was performed after the end of the sampling period, collecting 3× 15-min samples per participant, and used as the calibration method for microdialysis by calculation of relative recovery (RR). RR was used to calculate ISF concentrations from microdialysate concentrations. Assays and sampling preparation and storage have been described.^24^

### Pharmacokinetic model development and evaluation

Data from all sources (plasma, microdialysis, and retrodialysis) were analysed simultaneously for model development. The model was parameterised in terms of cefazolin unbound concentrations. Based on the PK insights of a published noncompartmental analysis (NCA) of these plasma and ISF data,^24^ for the compartmental nonlinear mixed-effects modelling approach, one-, two-, and three-compartment models were tested. Linear, saturable (nonlinear) and combined (linear plus saturable) plasma protein binding models were evaluated to characterise the relationship between total and unbound cefazolin concentrations. Microdialysis concentrations were analysed using the integrated dialysate-based approach.^25^^,^^26^

Interindividual variability in PK behaviour was evaluated on all model PK parameters. Inclusion of microdialysis intracatheter and intercatheter variability on RR was also evaluated. Additive, proportional, and combined residual variability models were tested to account for deviations between predictions and observed data.

To explain potential differences in cefazolin exposure between obese and nonobese patients, the impact of different body size descriptors on PK parameters (volume of distribution and flow) was evaluated: allometric scaling, with fixed and estimated exponents, based on total body weight (TBW), lean body weight (LBW),^27^ ideal body weight (IBW),^28^ adjusted body weight (ABW),^29^ and the LBW/fat mass (FM)^30^ and normal fat mass^31^ approaches were evaluated. The impact of clinical and participant characteristics on cefazolin clearance, estimated glomerular filtration rate, and age, was evaluated. The impact of obesity on protein binding parameters and RR was evaluated in a categorical way (different parameters for obese and nonobese patients).

Intermediate PK models were evaluated with standard goodness-of-fit plots (e.g. observed *vs* predicted concentrations and residuals *vs* population prediction and time). Predictive model performance was evaluated by visual predictive checks (*n*=1000), whereas the precision of the estimated parameters was assessed by sampling importance resampling.

### Dosing regimen simulation and evaluation

The developed PK model was leveraged to perform Monte Carlo simulations (*n*=1000). Simulations were performed for three reference patients: nonobese (TBW = 70 kg, FM = 24.8%, BMI = 24.2 kg m^−2^), obese (TBW = 95.1 kg, FM = 29.5%, BMI = 33.0 kg m^−2^), and morbidly obese (TBW = 127 kg, FM = 39.5%, BMI = 44.0 kg m^−2^). A minimum inhibitory concentration (MIC) ≤4 mg L^−1^ was chosen, which is the clinical breakpoint for nonresistant *Escherichia coli* and *Staphylococcus aureus* (EUCAST^32^). The PK/PD target used to evaluate the adequacy of therapy was unbound cefazolin concentration exceeding MIC 100% of the time (*f*T_>MIC_ = 100%) after 8 h.^19^ Dosing regimens were considered adequate for a reference patient when 90% of the simulated concentration–time profiles achieved this PK/PD target, specifically when the probability of target attainment (PTA) was ≥90%.^33^ To evaluate bacterial infection scenarios when the MIC is unknown, as for surgical prophylaxis, cumulative fraction of response (CFR),^34^ a weighted average of PTA over the MIC distribution of the pathogens of interest*,* was calculated. As with PTA analysis, dosing regimens were considered adequate when CFR was ≥90%.^33^ Four different dosing regimens (of dosing plus redosing) were simulated for each reference patient (30-min infusion for all administered doses): 1 g (redosing 1 g at 3 h), 1 g (redosing 1 g at 4 h), 2 g (redosing 2 g at 3 h), and 2 g (redosing 2 g at 4 h).

### Software

Modelling was performed in NONMEM v7.4.3 (Icon Development Solutions, Ellicott City, MD, USA). PsN (Perl Speaks NONMEM) v4.8.1 was used to access NONMEM through Pirana v2.9.6 (Certara, Princeton, NJ, USA). R v4.2.1 with RStudio (Boston, MA, USA) was used for data management, data visualisation, and processing of modelling results. Simulations were performed using mrgsolve R package v1.0.6.

## Results

Data were collected from 15 obese participants with a median BMI of 52.6 kg m^−2^ (range 39.5–69.3 kg m^−2^) and 15 age- and sex-matched nonobese patients with a median BMI of 26.0 kg m^−2^ (range 18.7–29.8 kg m^−2^). An overview of participant characteristics can be found in Table 1.

**Table 1:** Summary of participant characteristics. ABW, adjusted body weight; BMI, body mass index; eGFR, estimated glomerular filtration rate; FM, fat mass; IBW, ideal body weight; LBW, lean body weight; TBW, total body weight.

Participant characteristics	Nonobese (*n*=15)	Obese (*n*=15)	All (*n*=30)
Sex, *n* (%)			
Male	5 (33.3)	5 (33.3)	10 (33.3)
Female	10 (66.7)	10 (66.7)	20 (66.7)
Age (yr), median (range)	45 (21–65)	45 (25–65)	45 (21–65)
eGFR (CKDEPI) (ml min^−1^), median (range)	103.8 (86.1–121.7)	133 (82.8–269)	119 (82.8–269)
BMI (kg m^−2^), median (range)	26.0 (18.7–29.8)	52.6 (39.5–69.3)	34.7 (18.7–69.3)
TBW (kg), median (range)	78.0 (50.0–96.0)	155 (123–200)	109 (50.0–200)
LBW (kg), median (range)	45.6 (34.2–72.4)	69.3 (54.9–96.1)	64.3 (34.2–96.1)
ABW (kg), median (range)	59.4 (52.3–85.2)	102 (80.4–128)	81.0 (52.3–128)
IBW (kg), median (range)	59.4 (52.3–85.2)	63.9 (48.7–79.0)	61.2 (48.7–85.2)
FM (kg), median (range)	25.0 (15.8–34.2)	84.2 (48.8–122)	41.5 (15.8–122)

### Pharmacokinetic model

**Fig 1:**
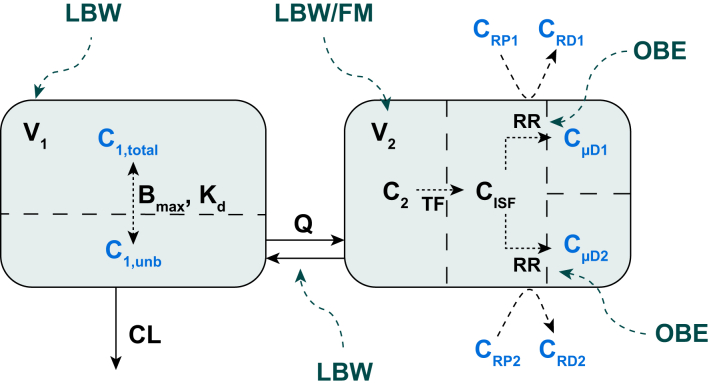
Model structure. Black: estimated parameters; blue: observed quantities; green: patient characteristics. B_max_, maximum binding capacity; C_1,total_, total concentration in the central compartment; C_1,unb_, concentration unbound in the central compartment; C_2_, concentration in the peripheral compartment; C_ISF_, interstitial space fluid concentration; CL, clearance; C_RD1,2_, retrodialysate concentration (catheters 1 and 2); C_RP1,2_, retroperfusate concentration (catheters 1 and 2); C_μD1,2_, microdialysate concentration (catheters 1 and 2); FM, fat mass; ISF, interstitial space fluid; K_d_, dissociation constant; LBW, lean body weight; OBE, obesity; Q, intercompartmental clearance; RR, relative recovery; TF, tissue factor; V_1_, central volume of distribution; V_2_, peripheral volume of distribution.

The final model was a two-compartment model with linear elimination (Fig. 1 and Table 2). Cefazolin protein binding was best characterised by a saturable binding model (Supplementary Fig. S1) with a maximum binding capacity of 247 mg L^−1^ (95% confidence interval [CI] 207–286 mg L^−1^) and a dissociation constant of 65.3 mg L^−1^ (95% CI 49.9–80.7 mg L^−1^), meaning that a maximum of 247 mg L^−1^ of cefazolin can be bound in plasma and that, at 65.3 mg L^−1^ unbound cefazolin, half of the binding partner sites are occupied. Microdialysate concentrations were attributed to the peripheral compartment and converted to ISF concentrations by estimated RR. A significant effect of obesity was found (RR_obese_ = 23.3%, 95% CI 18.6–28.0%; RR_nonobese_ = 41.1%, 95% CI 30.4–51.8%). Of note, differences in RR are microdialysis technique- and tissue-related. A tissue scaling factor (TF = 0.655, 95% CI 0.574–0.736) relating predicted concentrations in the peripheral compartment to ISF concentrations was identified, and was not different between obese and nonobese patients (Table 2).

**Table 2:** Summary of model parameter estimates for unbound cefazolin and precision by sampling importance resampling. μD, microdialysis; B_max_, maximum binding capacity; CI, confidence interval; CL, clearance; CV, coefficient of variation; FM, fat mass; K_d_, dissociation constant; LBW, lean body weight; Q, intercompartmental flow; R, scaling factor in the LBW/FM approach (fraction scaled with LBW); RD, retrodialysis; RR, relative recovery; SIR, sampling importance resampling; TF, tissue factor; V_1_, central volume of distribution; V_2_, peripheral volume of distribution. ∗Implementation of the LBW/FM approach. ^†^Fixed parameters.

Parameter (unit)	Parameter estimate	SIR 95% CI
**Fixed-effects parameters**		
CL (L h^−1^)	17.9	16.6–19.2
V_1_, (L) (LBW = 64.3 kg)∗	22.9	19.2–26.5
Exponent V_1_	1^†^	–
Q (L h^−1^) (LBW = 64.3 kg)∗	56.8	49.2–66.9
Exponent Q	0.75^†^	–
V_2_ (L) (LBW = 64.3 kg, FM = 41.5)∗	34.3	32.4–36.9
R (%)	76.4	64.9–86.0
B_max_ (mg L^−1^)	247	219–289
K_d_ (mg L^−1^)	65.3	55.8–79.9
TF (%)	65.5	59.4–70.9
RR_obese_ (%)	23.3	19.4–29.1
RR_nonobese_ (%)	41.1	35.3–48.8
**Interindividual variability, CV (%)**		
CL	21.9	17.9–29.0
V_1_	40.1	28.7–56.4
Q	54.8	42.0–72.9
V_2_	12.4	6.33–18.6
B_max_	9.20	5.66–12.9
RR	43.3	22.8–63.0
Intercatheter	58.6	42.6–80.7
Intracatheter	90.3	73.8–110
**Residual variability, CV (%)**		
Plasma proportional error	35.7	33.9–37.7
μD proportional error	52.1	50.4–54.0
RD proportional error	28.9	23.8–36.3

Interindividual variability was included on all PK parameters, except K_d_, and was low to moderate (coefficient of variation [CV] ≤54.8%). Furthermore, interindividual variability was included in RR (CV = 43.3%), as well as intracatheter and intercatheter variability (CV = 90.3% and 58.6%, respectively). Both plasma total and unbound microdialysis and retrodialysis data were best characterised by a proportional residual variability model.

Models with fixed exponents and estimated exponents for allometric scaling performed similarly. Fixed exponents were preferred because of their mechanistic foundations. Model performance (based on the Akaike information criterion) was similar between the LBW/FM^30^ approach and allometric scaling based on ABW^29^ (AIC_LBW/FM_ = 2500, AIC_ABW_ = 2494). The LBW/FM approach was chosen because of its physiologically motivated foundations and interpretability. The chosen model showed good predictive performance and characterised the data well (Supplementary Figs S2–S4). Based on the LBW/FM approach, 23.6% of cefazolin peripheral volume of distribution was found to depend on FM, whereas the remaining fraction (76.4%) depended on LBW. The central volume of distribution and intercompartmental flow scaled linearly with LBW. No clinical or participant characteristics were found to have an impact on cefazolin clearance (17.9 L h^−1^). No significant differences between obese and nonobese participants were found for protein binding.

### Dosing regimen simulation and evaluation

**Fig 2:**
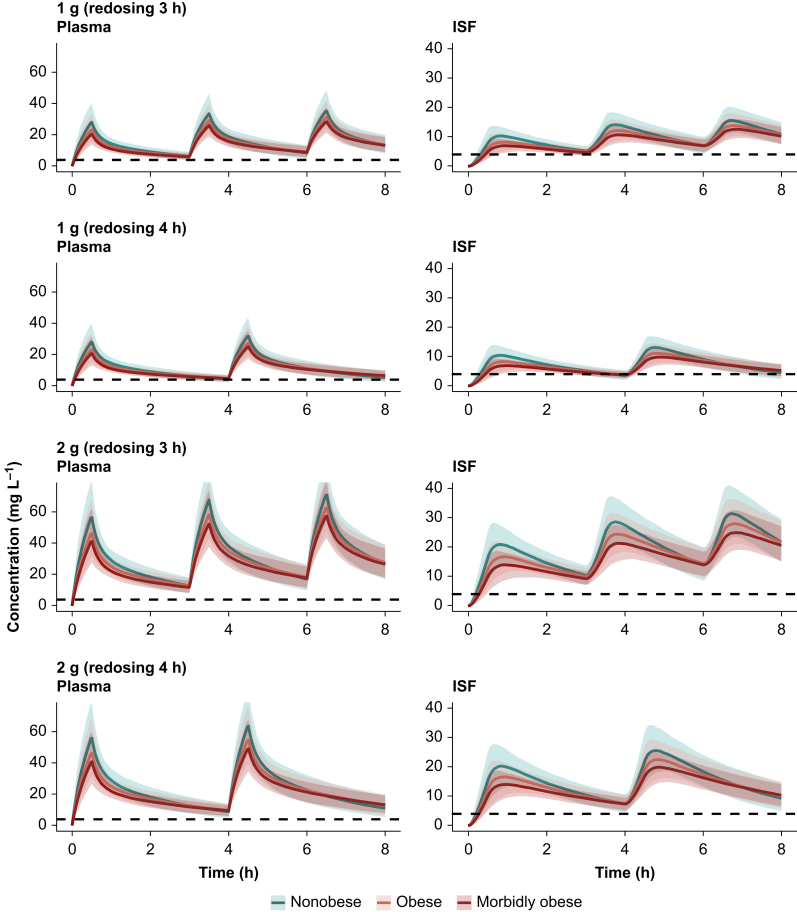
Simulated (*n*=1000) cefazolin concentration–time profiles for nonobese, obese, and morbidly obese reference patients. Coloured lines represent the median; shaded areas are 5th–95th percentiles; and the dashed black line represents MIC = 4 mg L^−1^. ISF, interstitial space fluid; MIC, minimum inhibitory concentration.

**Fig 3:**
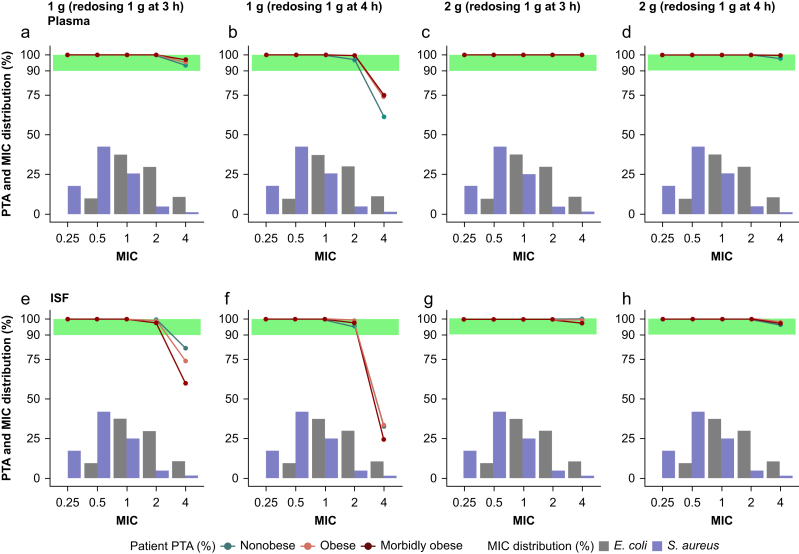
Probability of target attainment (≥90%) of unbound cefazolin in (a–d) plasma and (e–h) interstitial space fluid for four different dosing regimens: (a, e) 1 g with redosing after 3 h, (b, f) 1 g with redosing after 4 h, (c, g) 2 g with redosing after 3 h, and (d, h) 2 g with redosing after 4 h. Bars represent MIC distribution of *Escherichia coli* and *Staphylococcus aureus*. ISF, interstitial space fluid; MIC, minimum inhibitory concentration; PTA, probability of target attainment.

To evaluate the need for dosing adjustments in obese patients, unbound plasma and ISF concentration–time profiles were simulated for nonobese, obese, and morbidly obese reference patients (Fig. 2). Probability of target attainment analysis was performed evaluating up to MIC = 4 mg L^−1^, the clinical breakpoint for cefazolin for both *E. coli* and *S. aureus* (EUCAST^32^). Overall, PTA was found to be similar in nonobese reference patients compared with obese and morbidly obese reference patients. When the MIC was ≤2 mg L^−1^, all four evaluated dosing regimens were adequate for both unbound plasma and ISF (Fig. 3) PTA evaluation. However, different results were obtained when evaluating PTA in plasma compared with ISF with MIC = 4 mg L^−1^. In particular, 1 g (redosing 1 g at 3 h) and 2 g (redosing 2 g at 4 or 3 h) were found to be adequate dosing regimens (PTA ≥90%) for all three reference patients in plasma, whereas 1 g (redosing 1 g at 4 h) was found to be inadequate (PTA <90%) (Fig. 3b) (PTA was 60.9% in nonobese, 73.4% in obese, and 74.5% in morbidly obese). However, when PTA was evaluated in ISF, 1 g (redosing 1 g at 3 h) (Fig. 3e) was found inadequate for all three reference patients (PTA was 85.5% in nonobese, 75.1% in obese, and 61.2% in morbidly obese).

**Fig 4:**
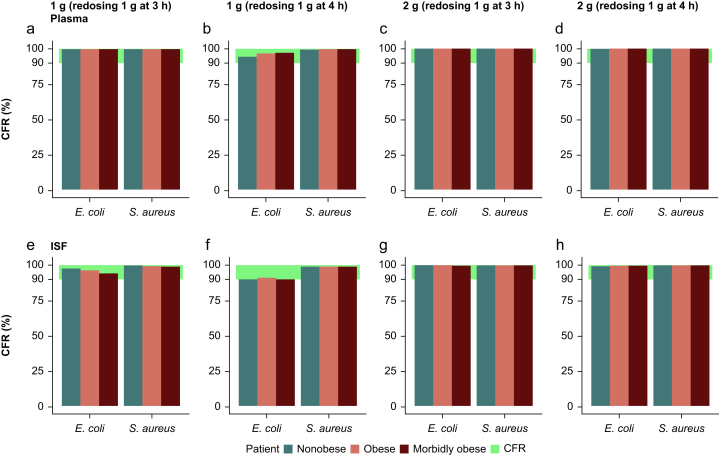
Cumulative fraction of response (≥90%) of unbound cefazolin in (a–d) plasma and (e–h) interstitial space fluid for different dosing regimens: (a, e) 1 g with redosing after 3 h, (b, f) 1 g with redosing after 4 h (c, g) 2 g with redosing after 3 h and (d, h) 2 g with redosing after 4 h in nonobese, obese, and morbidly obese reference patients. CFR, cumulative fraction of response; ISF, interstitial space fluid.

Similar to PTA analysis, CFR analysis did not show different results between nonobese, obese, and morbidly obese reference patients (Fig. 4). All evaluated dosing regimens reached CFR ≥90% for all three reference patients in both plasma and ISF.

No differences in PTA and CFR results between nonobese and obese patients were observed under any of the evaluated scenarios. Based on the performed PTA and CFR analyses, 2 g (redosing 2 g at 4 h) appeared as the most suitable dosing regimen when MIC is unknown or MIC = 4 mg L^−1^. However, in the case of known MIC ≤2 mg L^−1^, 1 g (redosing 1 g at 4 h) was also found to be an adequate dosing regimen. Ultimately, no benefit of redosing at 3 h compared with 4 h was observed under any of the evaluated dosing regimen scenarios for all three reference patients.

## Discussion

The need for more than 2 g doses of cefazolin in obese patients for perioperative antibiotic prophylaxis has been studied and debated with differing results. We evaluated the adequacy of clinically relevant cefazolin dosing regimens for surgical prophylaxis based on exposure in plasma and ISF of adipose tissue, the usual site of infection. Using *f*_T>MIC_ = 100% as a target, we did not find any clinically relevant influence of obesity. A second dose after 4 h was sufficient in the context of perioperative antibiotic prophylaxis with an administration of 2 g.

The data presented in this study were previously published and analysed using noncompartmental analysis (NCA).^24^ The NLME approach of this study confirmed the NCA results revealing no clinically relevant differences in cefazolin concentrations between obese and nonobese patients. Based on the evaluated scenarios, our results agree with the majority of PK and outcome studies conducted^12^^,^^35^, ^36^, ^37^, ^38^ to investigate the need for a higher cefazolin dose in obese patients, concluding it is not needed. Based on these results, the higher incidence of SSIs observed in obese patients does not appear related to inadequate cefazolin dosing regimens. The large range in patient BMI (18.7–69.3 kg m^−2^) included in this study allowed us to characterise PK differences between obese and nonobese patients based on differences in body composition. However, these PK differences were not considered to be clinically relevant in terms of dosing regimen adequacy. However, because a higher incidence of SSIs and lower plasma and ISF concentrations are observed in obese patients early after dosing, whether time-related targets are not the best efficacy measure must be considered and could be explored in future studies.

Although this analysis agrees with previous findings, it further suggests that doses lower than 2 g could be sufficient. However, practically, because the MIC cannot be established in a prophylactic setting, 2 g remains the recommended dose for obese and nonobese patients. Nonetheless, in a local setting when the MIC distribution is known, the same alternative doses might be considered for both obese and nonobese patients. One modelling and simulation study that concluded that obese patients require a dose adaptation did not include a control group (nonobese) and the majority of samples were collected only up to 2.5 h after dosing,^13^ whereas the other two included only pregnant women as obese population^14^ or included a low number of patients (*n*=15).^15^ These differences from the present study design and study population might explain the different conclusions obtained.

For perioperative antibiotic prophylaxis, it is typically recommended to redose after twice the half-life of the antibiotic. However, because the reported half-life for cefazolin ranges between 1.5 and 2 h, redosing at 3 and 4 h was evaluated in this study. No benefits of redosing after 3 h were observed compared with redosing at 4 h at a dose of 2 g. Therefore, for practical reasons, redosing is recommended 4 h after the first cefazolin administration for longer surgical procedures.

We acknowledge that the sample size of patients included in the study (*n*=30) is a limitation. However, it has to be considered a large sample when compared with other microdialysis studies. Because systemic and target-site exposure might differ,^20^^,^^21^ being able to perform such evaluations at the probable site of infection is of great value.

Saturable binding of cefazolin was observed and quantified, consistent with previous studies.^15^^,^^39^^,^^40^ However, albumin is considered the main binding partner for cefazolin, and its concentration is much higher than the maximum binding capacity estimated in this study. Nevertheless, the nonlinear binding behaviour has been observed consistently, suggesting that the binding mechanisms remain unclear.

No impact of participant or clinical characteristics was included for cefazolin clearance. As cefazolin is mostly eliminated renally, an impact of estimated glomerular filtration rate (eGFR) on clearance could be expected. All eGFR calculation methods point towards increased renal function for obese individuals; however, obesity is a risk factor for chronic kidney disease.^7^ Therefore, identification of eGFR to explain expected clearance differences between obese and nonobese patients might be nontrivial. To overcome this, in future studies, measurements of GFR should be performed. Lastly, although use of PK/PD targets is well established to link drug exposure to efficacy, data on clinically measured outcome endpoints should, in the future, be collected, at least in observational settings.

In conclusion, with the evaluated condition, this study showed that in the context of perioperative antibiotic prophylaxis with cefazolin, no dose adjustment is necessary in obese patients. In general, initial administration of 2 g cefazolin i.v. with redosing at 4 h intraoperatively is sufficient.

## Authors’ contributions

Conception of study design: DBi, PS, DBu, MZ, HW, CK

Study design: DBi, PS, DBu, DP, CK

Patient recruitment: PS, DP, HW

Data collection: DBi, PS, DP, CD, HW

Data analysis: DBi, PS, DBu, DP, CD

Drafting the manuscript: DBi, PS

Critical revision for important intellectual content: all authors

## Declarations of interest

DBi, DBu, RM, and LA have no conflict of interest. CK and WH report grants from an industry consortium (AbbVie Deutschland GmbH & Co. K.G., Astra Zeneca, Boehringer Ingelheim Pharma GmbH & Co. K.G., F. Hoffmann-La Roche Ltd., Merck KGaA, Novo Nordisk, and Sanofi) for the PharMetrX PhD programme (Berlin/Potsdam, Germany). CK reports an additional grant from the Innovative Medicines Initiative-Joint Undertaking (‘DDMoRe’) and grants from the Federal Ministry of Education and Research within the Joint Programming Initiative on Antimicrobial Resistance Initiative (JPIAMR) and from the European Commission within in the Horizon 2020 framework programme (‘FAIR’), all outside the submitted work. HW and PS received grants from Pfizer (Investigator Initiated Trial Program, Berlin, Germany) and InfectoPharm (Heppenheim, Germany), both for the clinical microdialysis trials. PS reports lecture fees from InfectoPharm (Heppenheim, Germany). HW received lecture fees from Arjo (Mainz-Kastel, Germany).
