# Supplementary material for: Evaluation of the need for dosing adaptations in obese patients for surgical antibiotic prophylaxis: a model-based analysis of cefazolin pharmacokinetics
Source: Br J Anaesth. 2025 Feb 1;134(4):1041–9. doi: 10.1016/j.bja.2024.11.044 (PMC11947583; doi:10.1016/j.bja.2024.11.044)
Supplement: Multimedia component 1 [file mmc1.docx]

**Figures**


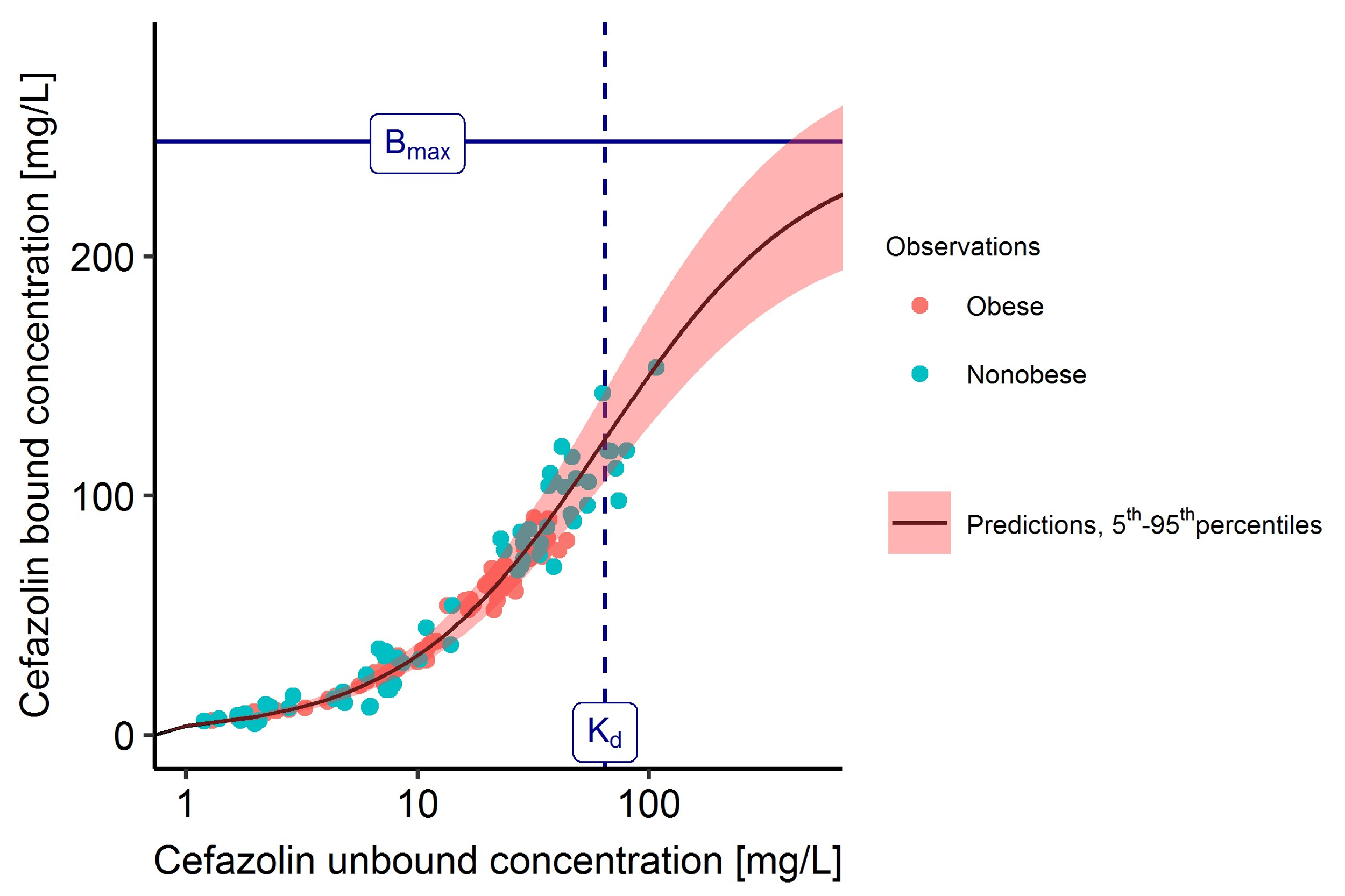


Figure S1 Relationship between cefazolin bound and unbound concentrations. Dots: Observations, Line and shaded area: Predictions and 90% confidence interval. Horizontal blue line: Maximum binding capacity (Bmax), vertical blue dashed line: Dissociation constant (Kd)


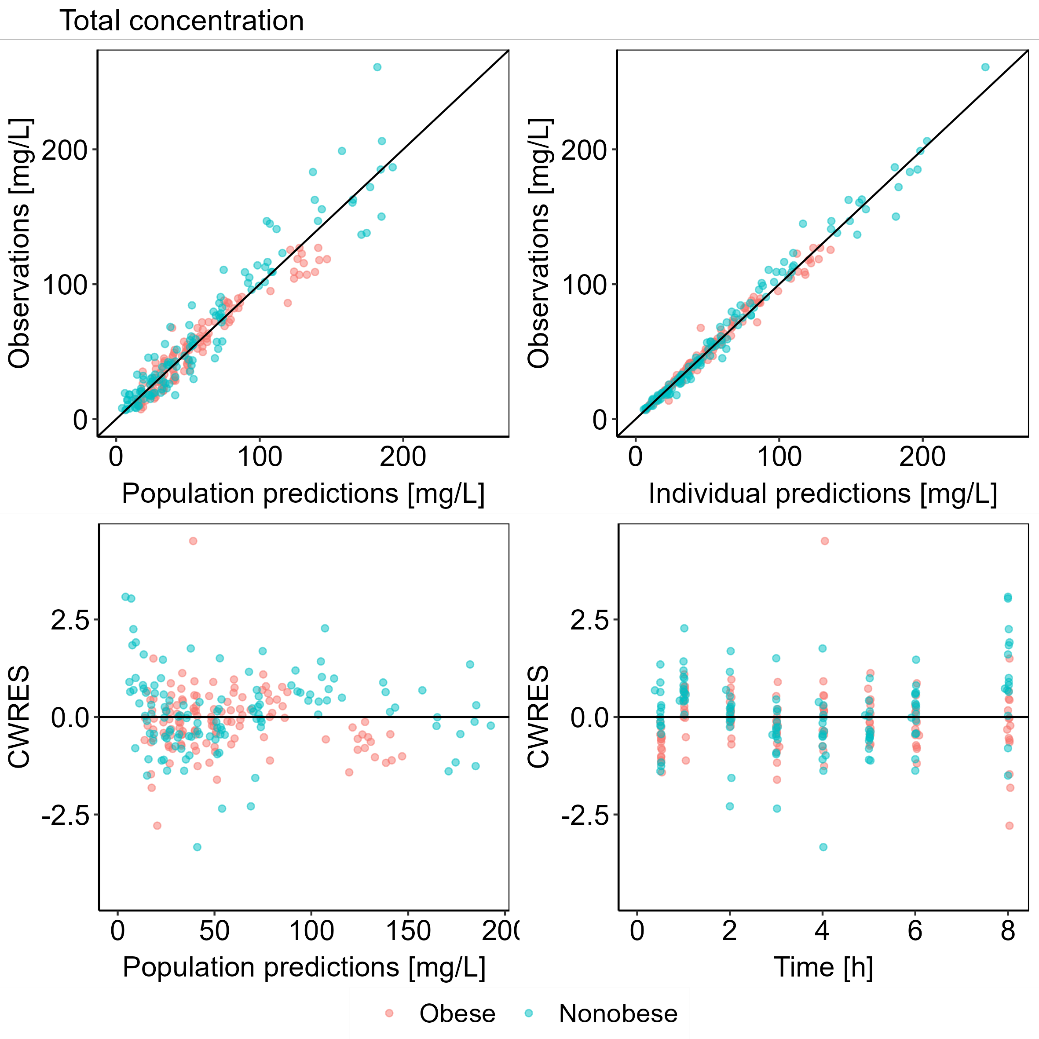

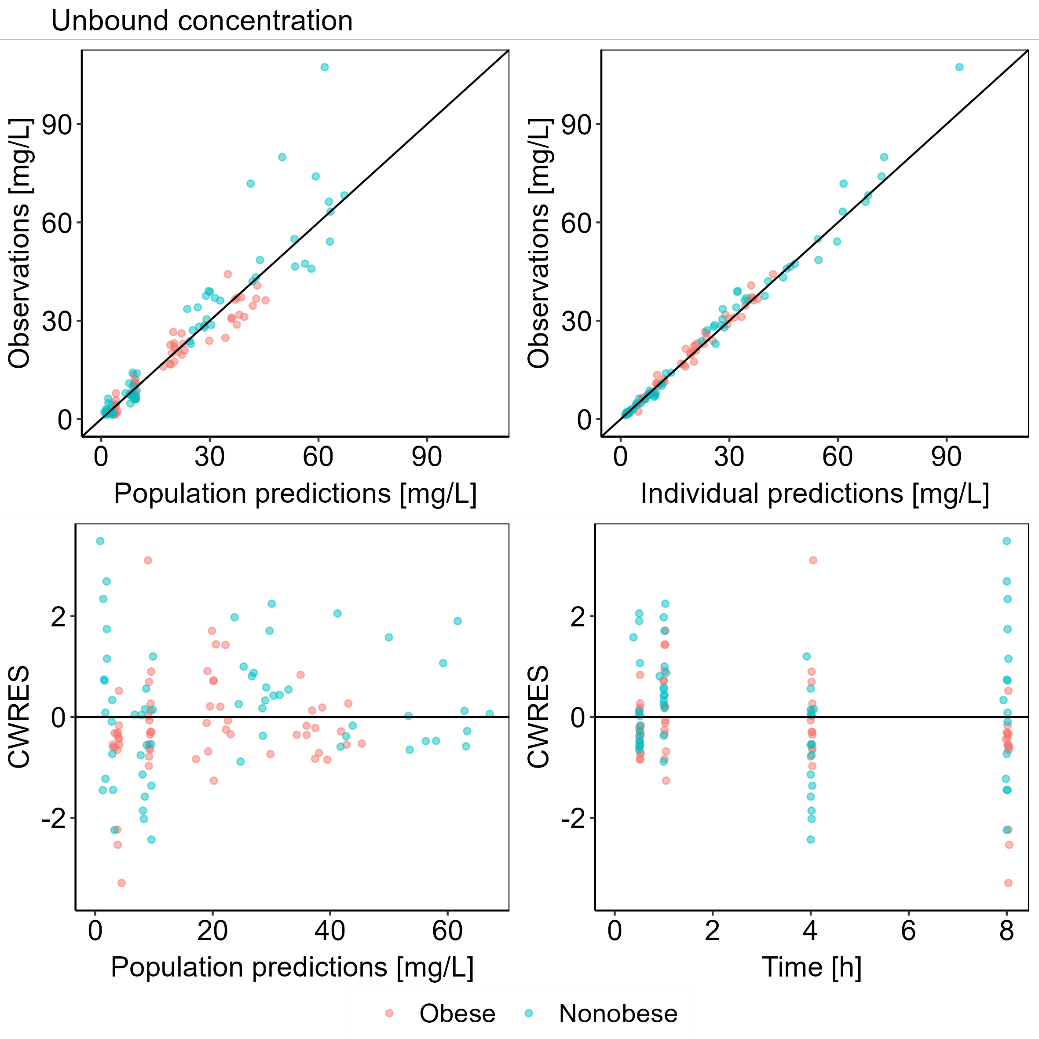


Figure S2 Goodness of fit plots for total and unbound cefazolin plasma concentration, and microdialysate and retrodialysate cefazolin concentration


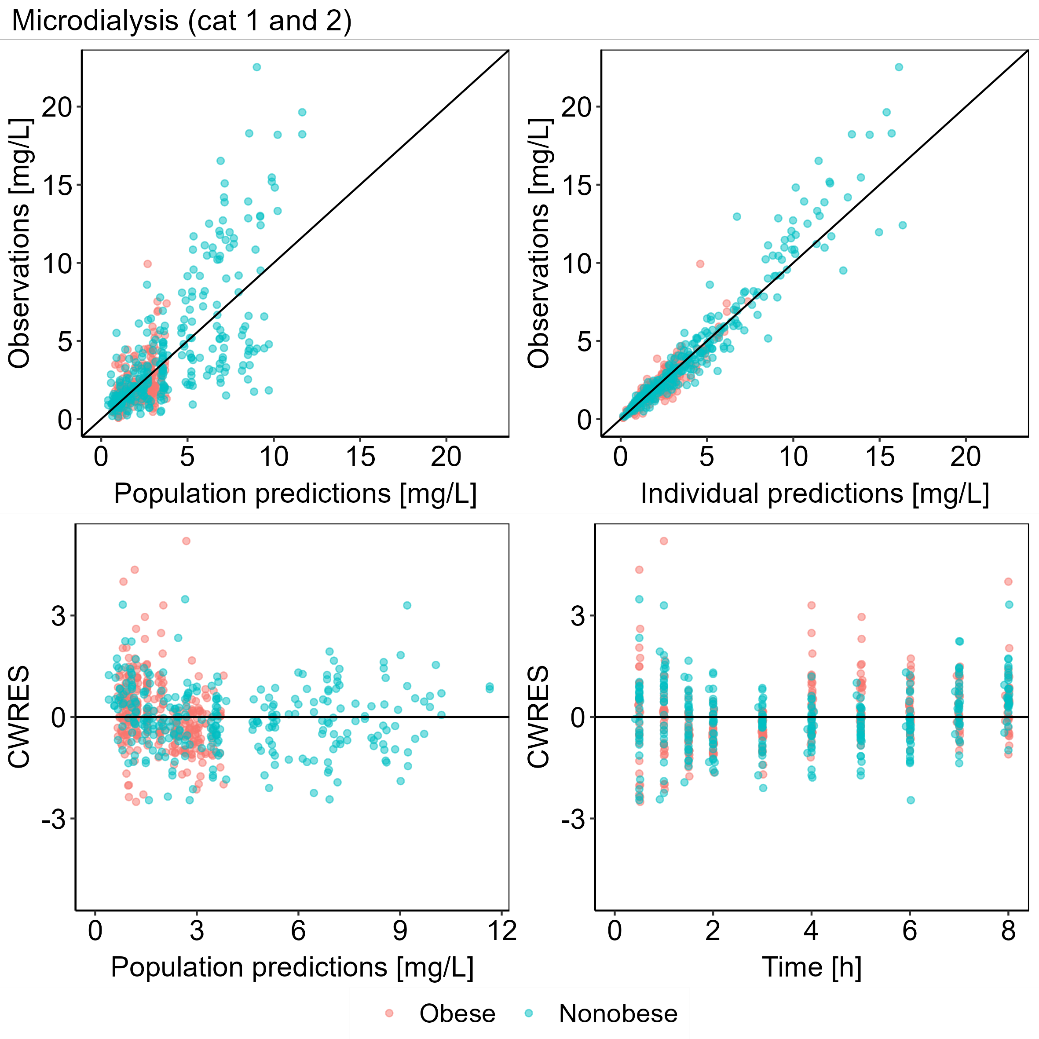

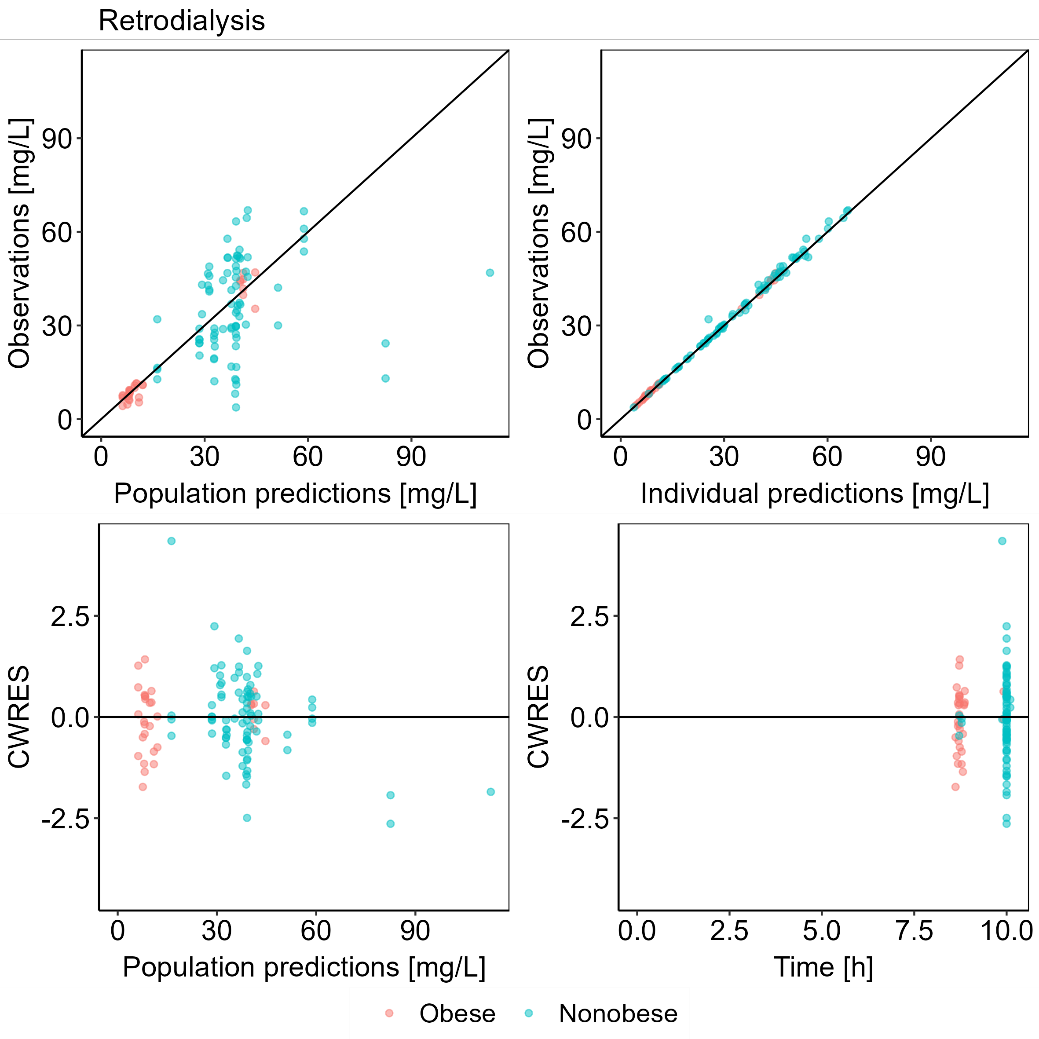


**
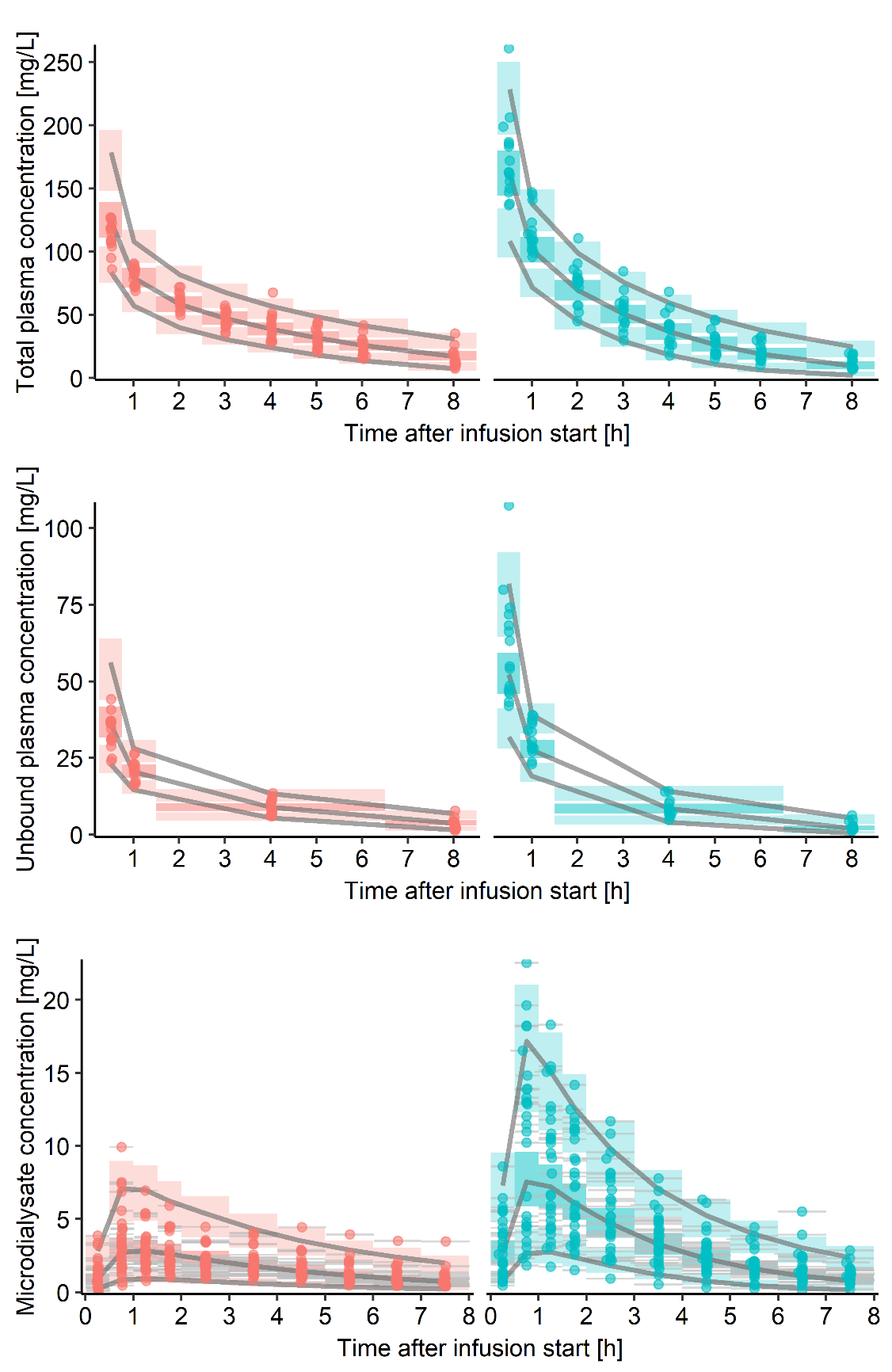
**

Figure S3 Visual predictive check for obese (red, left panels) and nonobese (cyan, right panels) patient. Gray lines: median and 90% prediction interval, coloured boxes: 90% confidence interval, points: Observations

**
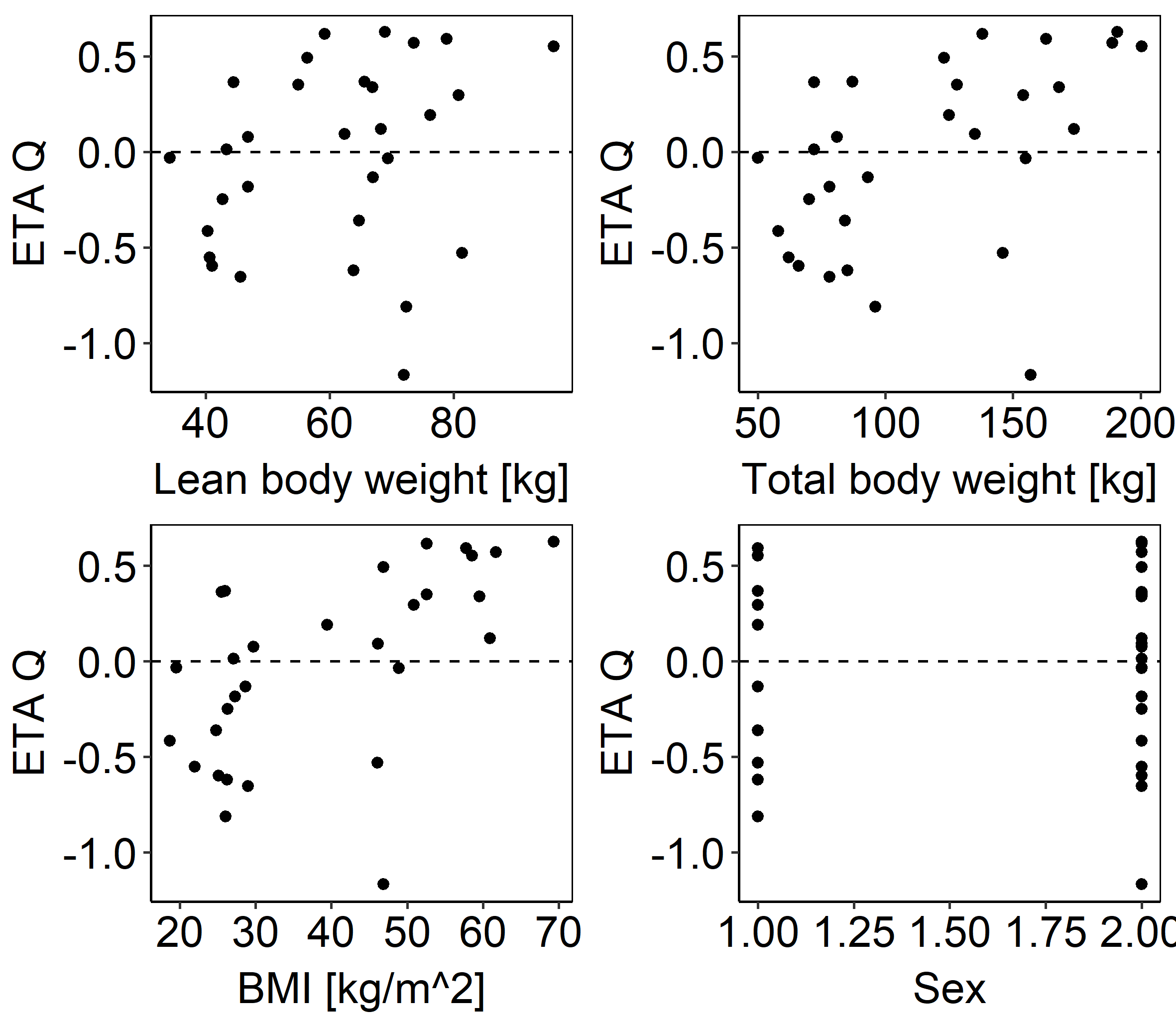

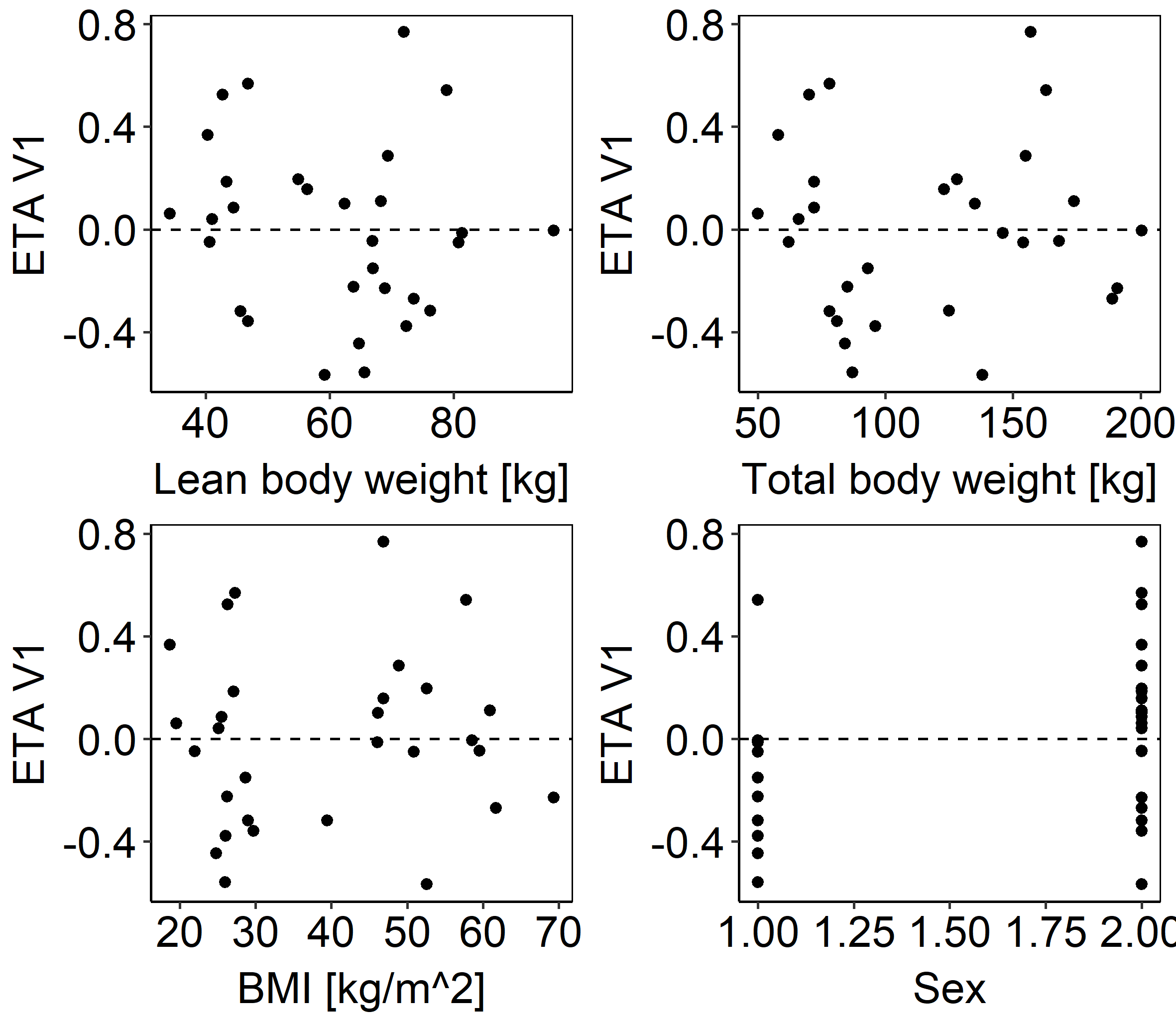

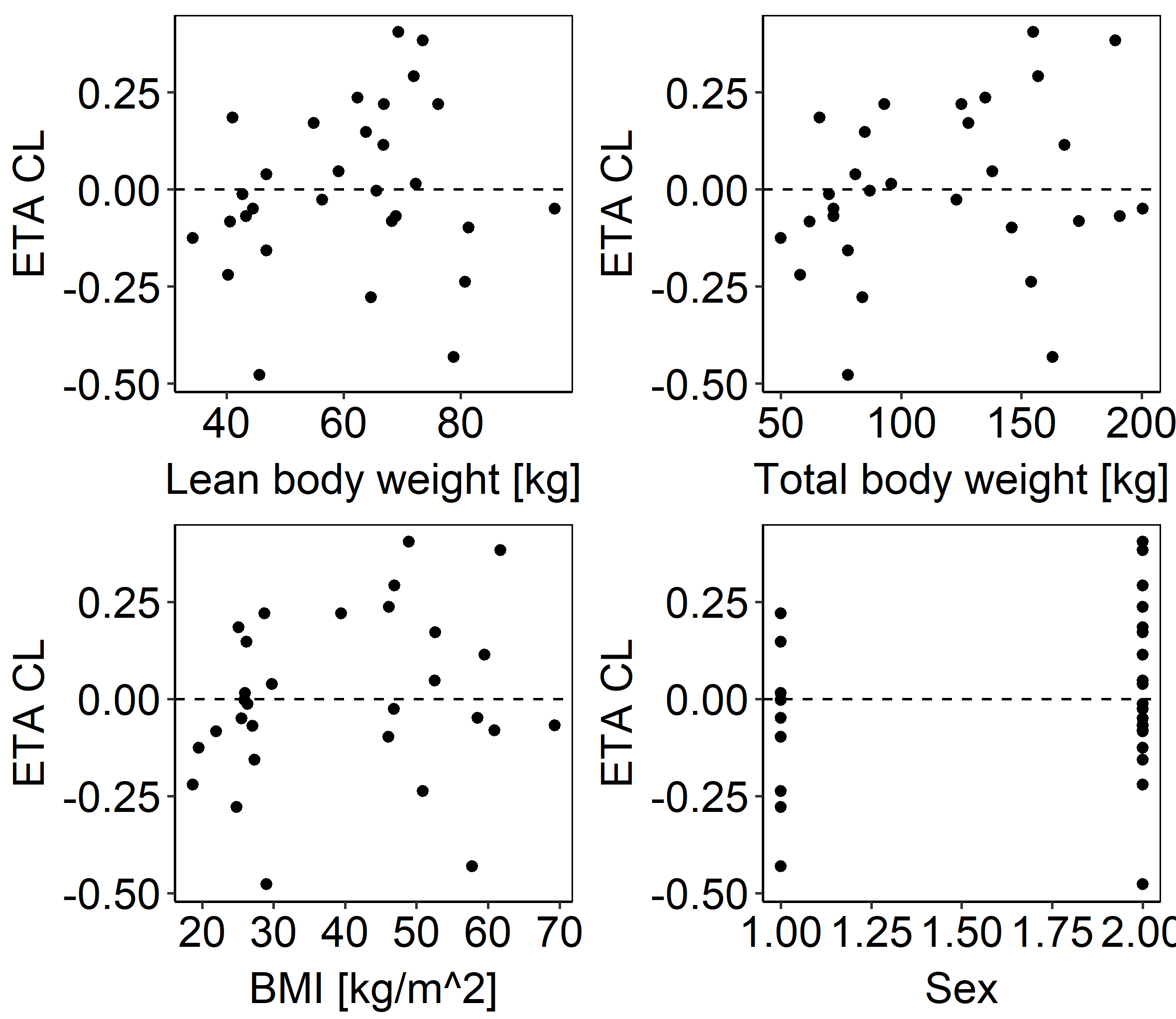
**

**
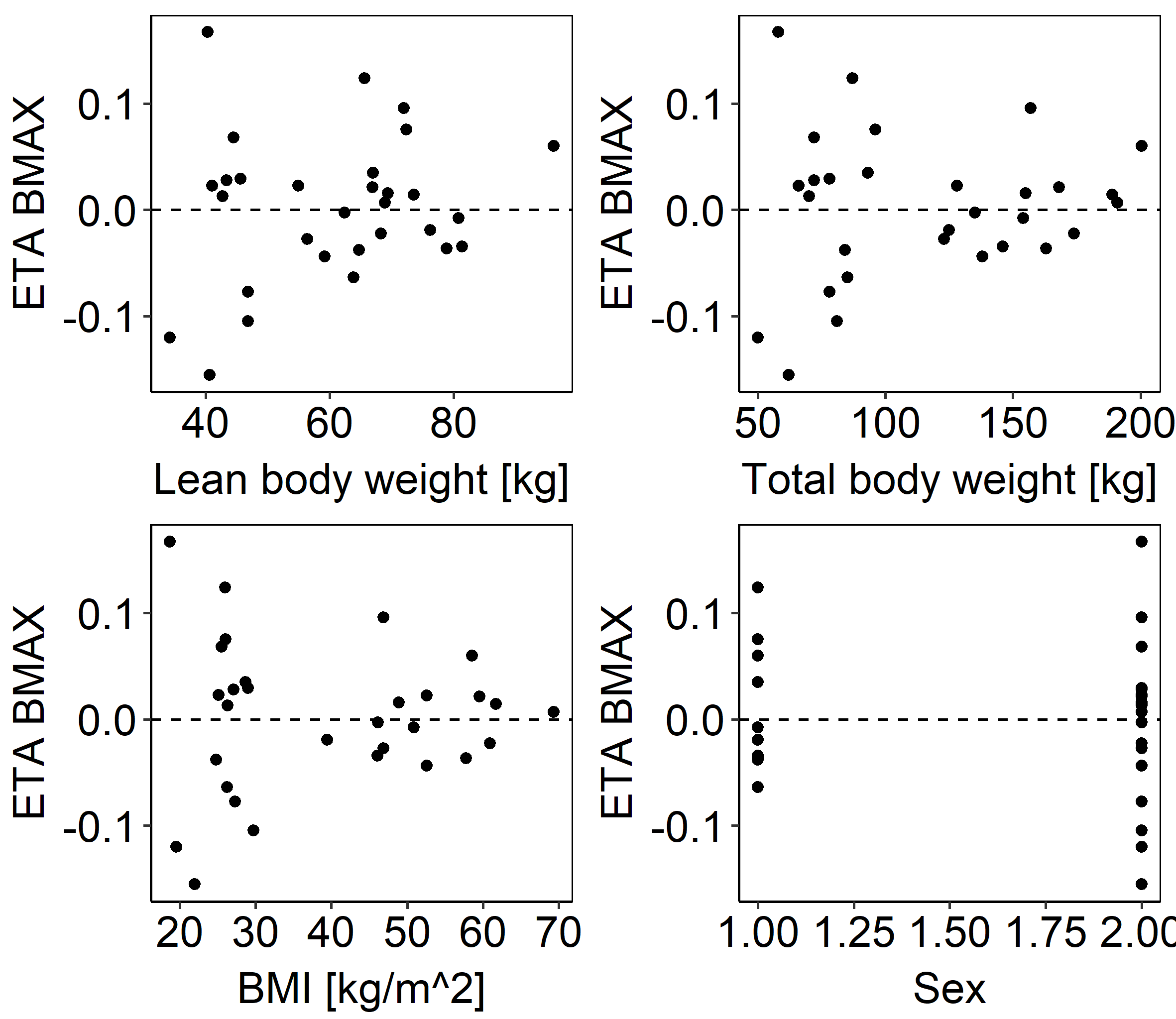

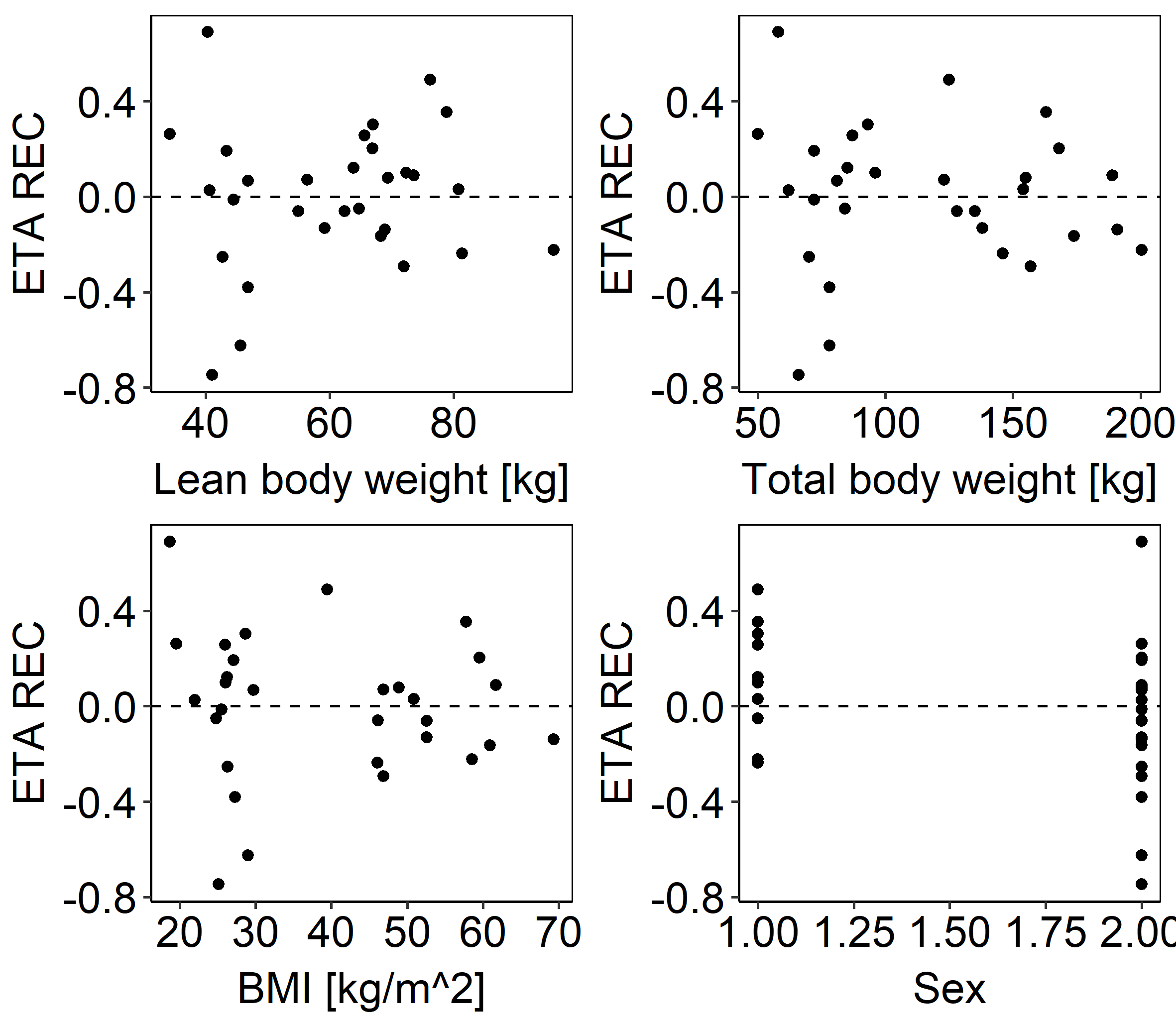

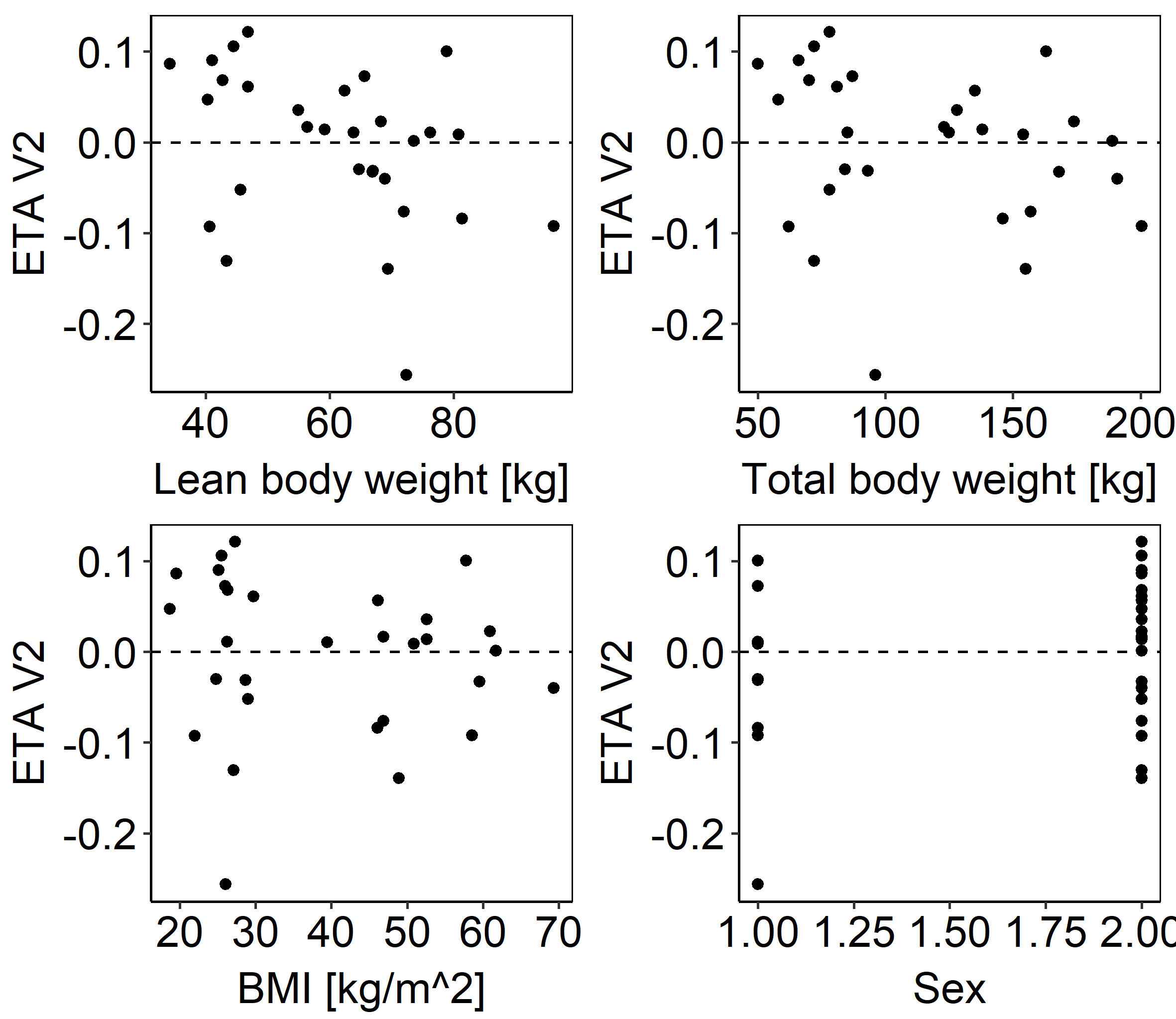
**

Figure S4 ETA plots for clearance (CL), volume of distribution (V1), intercompartmental flow (Q), peripheral volume of distribution (V2), relative recovery (REC), maximum binding capacity (BMAX) versus covariates.
BMI: Body mass index

**Tables**

Table S1 Summary for the comparison of different allometric scaling models: Theory-based allometric scaling using different body size descriptors and semi-mechanistic approaches (LBW/FM, NFM)

| **Approach/BSD** | **AIC** | **RSE (%), fixed effects** |
| --- | --- | --- |
| TBW* | 2508 | ≤11% |
| IBW* | 2541 | ≤17% |
| LBW* | 2509 | ≤12% |
| ABW* | 2494 | ≤12% |
| LBW/FM | 2500 | ≤11% |
| NFM | 2498 | ≤96% |
| *Theory-based allometric scaling | | |

**Model code for simulations**

https://github.com/Kloft-Lab/Bindellini-et-al._CefazolinPK_Obese_Nonobese_mrgsolve_model
